# Increasing the bactofection capacity of a mammalian expression vector by removal of the f1 *ori*

**DOI:** 10.1038/s41417-018-0039-9

**Published:** 2018-08-13

**Authors:** Síle A. Johnson, Michael J. Ormsby, Anne McIntosh, Stephen W. G. Tait, Karen Blyth, Daniel M. Wall

**Affiliations:** 1 Institute of Infection, Immunity and Inflammation, College of Medical, Veterinary and Life Sciences, Sir Graeme Davies Building, University of Glasgow, Glasgow, G12 8TA UK; 2 Cancer Research UK Beatson Institute, University of Glasgow, Garscube Estate, Switchback Road, Glasgow, G61 1BD UK

**Keywords:** Targeted therapies, Transfection, Genetic vectors

## Abstract

Bacterial-mediated cancer therapy has shown great promise in in vivo tumour models with increased survival rates post-bacterial treatment. Improving efficiency of bacterial-mediated tumour regression has focused on controlling and exacerbating bacterial cytotoxicity towards tumours. One mechanism that has been used to carry this out is the process of bactofection where post-invasion, bacteria deliver plasmid-borne mammalian genes into target cells for expression. Here we utilised the cancer-targeting *Salmonella* Typhimurium strain, SL7207, to carry out bactofection into triple negative breast cancer MDA-MB-231 cells. However, we noted that post-transformation with the commonly used mammalian expression vector pEGFP, *S*. Typhimurium became filamentous, attenuated and unable to invade target cells efficiently. Filamentation did not occur in *Escherichia coli*-transformed with the same plasmid. Further investigation identified the region inducing *S*. Typhimurium filamentation as being the f1 origin of replication (f1 *ori*), an artefact of historic use of mammalian plasmids for single stranded DNA production. Other f1 *ori*-containing plasmids also induced the attenuated phenotype, while removal of the f1 *ori* from pEGFP restored *S*. Typhimurium virulence and increased the bactofection capacity. This work has implications for interpretation of prior bactofection studies employing f1 *ori*-containing plasmids in *S*. Typhimurium, while also indicating that future use of *S*. Typhimurium in targeting tumours should avoid the use of these plasmids.

## Introduction

*Salmonella enterica* serovar Typhimurium is a Gram-negative facultative intracellular pathogen that can cause diseases ranging from gastroenteritis to systemic infection. Infection is driven by a number of pathogenicity islands bearing virulence factors which are delivered into host cells by type three secretion systems borne on the same islands [1, 2]. *S*. Typhimurium is known to target tumours during infection and is capable of tumour growth arrest in in vivo tumour models [3–5]. Tumours offer bacteria a unique niche in which to grow, with nutrient availability high and with tumours being immune privileged sites offering protection to infiltrating bacteria [6, 7]. Bacteria are also attractive tumour targeting agents with a number of unique features including; capability for systemic administration, broad tumour specificity, immune activation at tumour sites, antibiotic sensitivity to allow easy removal and tumour cell-specific delivery of either DNA or protein of interest [8]. Previous efforts have sought to enhance the innate tumouricidal capabilities of bacteria through a variety of strategies. These have included the *S*. Typhimurium-mediated delivery of apoptotic proteins to tumour cells or overexpression of plasmid-encoded *Vibrio vulnificus* FlaB to slow tumour growth [9, 10]. It has also been reported that bacteria can be utilised for the delivery of small hairpin RNA (shRNA) and eukaryotic expression plasmids to cancer cells [11–13]. This latter strategy, termed bactofection [14, 15], utilises bacteria to deliver genetic material to a target cell or tissue and has been tested in a variety of cancer models [16–18], as well as other diseases such as cystic fibrosis [19, 20], colitis [21] and Leishmaniasis [22], or simply just for vaccination [23–26]. Once internalised, bacteria lyse releasing the plasmid for heterologous expression of the target protein by host mammalian cells [27]. The process is not restricted to phagocytic cells and there have been reports of extracellular bacteria mediating DNA transfer to host cells via conjugation [28]. The mechanisms underlying mammalian cell uptake and expression of delivered DNA remain incompletely understood but certain features of plasmids used for bactofection are thought to contribute to the success and efficiency of the process [29–31]. Alternative means of using bacteria for delivery of DNA into human cells are also under investigation, such as delivery through bacterial Type IV secretion systems, and these have also shown significant promise [32].

Multiple bacterial genera are now known to be capable of bactofection including *Escherichia, Listeria*, and *Salmonella* [13–36]. Previous studies utilising *Salmonella* spp. to deliver plasmids to cancer cells have used pro-apoptotic, as well as immunogenic genes to enhance the tumouricidal effects of the bacteria [11, 37, 38]. Many cancer cells produce cancer cell-specific de novo antigens, and thus act as cancer-specific signals for immune cells to target [39]. Bacteria have been used to exploit this by delivering eukaryotic expression vectors encoding tumour antigens to eukaryotic cells and this has also been employed for the purposes of vaccination against tumour cell challenge [36, 40].

Here, using the attenuated strain SL7207 [41, 42], we determined that plasmid carriage by *S*. Typhimurium tumour-targeting strains induced a filamentous phenotype, reducing invasion of cancer cells and subsequently bactofection. We determined that this phenotype was dependent on the presence of an f1 origin of replication (f1 *ori*) in the plasmid and that its removal eliminated the filamentous phenotype and restored invasion and bactofection of cancer cells. This work will have important implications for use of *Salmonella* in future bactofection studies. These f1 *ori*-containing plasmids are some of the most commonly used plasmids for the purposes of bactofection and mammalian DNA carriage by bacteria. The data presented here argues against their use in future studies of bacterial-mediated cancer therapy.

## Materials and methods

### Bacterial strains, plasmids and cancer cell lines

Bacterial strains used in this study are listed in Table 1. Bacteria were grown in Lysogeny broth (LB) supplemented with antibiotics at the following concentrations: kanamycin, 50 μg/ml; ampicillin, 100 μg/ml; chloramphenicol, 500 μg/ml or erythromycin, 50 μg/ml. Plasmids used in this study are detailed in Table 2. Electroporation was performed using an Eppendorf Eporator (1.75 Kv, 5 ms). MDA-MB-231 cells were obtained from the American Type Culture Collection. MDA-MB-231 cells were maintained in Roswell Park Memorial Institute (RPMI)-1640 media (Gibco®, 12633) supplemented with 10% foetal calf serum (FCS), 1 mM l-glutamine, 2 mM sodium pyruvate and 100 international units (IU)/ml penicillin/streptomycin (all Sigma) at 37 °C and 5% CO_2_. Cells were routinely tested using the MycoAlert PLUS *Mycoplasma* detection kit (Lonza) to ensure they were *Mycoplasma* free.

**Table 1:** Bacterial strains used in this study

Strain	Relevant genotype	Source
VNP20009	*S*. Typhimurium Δ*purA* Δ*msbB*	Dr John Pawelek (Yale University) [64]
SL7207	*S*. Typhimurium Δ*aroA*	Dr Siegfried Weiss (Helmholtz Centre for Infection Research) [41]
LT2	Laboratory *S*. Typhimurium strain	Dr Gillian Douce (University of Glasgow)
SL1344	*hisG* mutant of 4/74	Prof. Beth McCormick (Uni. of Massachusetts Medical School) [42]
JH3010	SL1344-*prgH-gfp*	Prof. Jay Hinton (University of Liverpool) [48]
JH3016	SL1344-*rpsM-gfp*	Prof. Jay Hinton (University of Liverpool) [48]
K12	Laboratory *E. coli* strain	Prof. Andrew Roe (University of Glasgow)
LF82	Adherent-Invasive *E. coli*	Prof. Daniel Walker (University of Glasgow)
F18	Commensal *E. coli*	Prof. Beth McCormick (Uni. of Massachusetts Medical School) [65]
DH5α	Laboratory *E. coli* strain	—
BL21	Laboratory *E. coli* strain	—

**Table 2:** Plasmids used in this study

Plasmid	Function	Features	Source
pEGFP-C2	Eukaryotic expression vector	*EGFP* under the control of the CMV promoter	Clontech
pUC19	High copy number plasmid	pUC19 origin of replication	NEB
	Source of *lacZ* gene	*lacZ* gene	
pLuc	Eukaryotic expression vector similar to pEGFP	Ampicillin resistance, *luciferase* transgene, f1 *ori*	Addgene, 45968
p*rpsM-GFP*	Prokaryotic GFP reporter plasmid	Constitutive prokaryotic GFP expression	[66]
pEGFP(-f1)	Test if removal of f1 *ori* from pEGFP abrogates filamentation	pEGFP lacking f1 *ori*	This study
pACYC-EGFP	Test if *EGFP* can drive filamentation in pACYC184	rpsmGFP plasmid plus EGFP transgene from pEGFP	This study

### Generation of the pACYC-EGFP and pEGFP(-f1) plasmids

pACYC-EGFP: *Escherichia coli* DH5α carrying pACYC184 were grown overnight in 10 ml LB supplemented with chloramphenicol and the plasmid isolated using a QIAprep® Spin Miniprep Kit (Qiagen). The *EGFP* gene was PCR amplified from pEGFP-C2, hereafter referred to as pEGFP, using the Q5 High-Fidelity DNA Polymerase kit (NEB). All primers used in this study are listed in Table 3. pACYC184 was digested with *Ase*I and *Sph*I (ThermoFisher Scientific) to generate a 1.5 kb fragment. Ligation of *EGFP* into the pACYC184 backbone was carried out using T4 DNA Ligase (NEB) per the manufacturers’ instructions before being transformed into competent *E. coli* DH5α cells.

**Table 3:** Primers used in this study

Primer name	Purpose	Sequence
Plasmid: pACYC-EGFP		
*EGFP* F	Amplification of CMV-EGFP	CTGCATTAATGCGTTACATAACTTACGGTAAATGG
*EGFP* R	Amplification of CMV-EGFP	CGACGCATGCACGCGTTAAGATACATTGATGAGTT
Plasmid: pEGFP(-f1)		
pEGFP(-f1) F	Amplification of vector backbone	CTGGGGTGCCTAATGAGTGATTTTATGTTTCAGGTTCAGGGG
pEGFP(f1-*ori*) R	Amplification of vector backbone	GGTTTTCACCGTCATCACCGCAATTAGTCAGCAACCAGGTG
*lacZ* F	Amplification of insert	TCACTCATTAGGCACCCCAG
*lacZ* R	Amplification of insert	CGGTGATGACGGTGAAAAC

pEGFP(-f1): Fragments were amplified by PCR from pEGFP (vector) and pUC19 (*lacZ* insert) as described above, using primers listed in Table 3. The primers were designed to provide overlapping sequences between the amplified products to promote homologous recombination upon ligation. The fragments (0.03–0.2 pmol at a vector: insert ratio of 1:2) were co-incubated in the presence of 1× NEBuilder® HiFi DNA Assembly Master Mix buffer (NEB) at 50 °C for 15 min to allow for plasmid assembly. Samples were then placed on ice prior to electroporation into competent *E. coli* BL21. Transformants containing the assembled plasmid were selected for on the appropriate antibiotic-containing LB agar and sequencing analysis used to confirm successful assembly.

### Bacterial growth, cell culture and infection

Bacteria were grown overnight with aeration in LB at 37  °C with shaking at 180 revolutions per minute (rpm). Bacteria were then back-diluted to an optical density at 600 nm (OD_600_) of 0.05 in 50 ml of LB culture and supplemented with antibiotics where appropriate. For growth curves, cultures were then allowed to grow as before with OD_600_ readings taken at regular intervals. For infection of mammalian cells, cultures were harvested at mid-log phase at an OD_600_ of ~0.6 and diluted in RPMI (3% FCS, 1% l-glutamine) to give a multiplicity of infection (MOI) of 100.

MDA-MB-231 cells were seeded at 5 × 10^5^ cells/well of a 6-well plate in RPMI (3% FCS, 1% l-glutamine) 24 h prior to infection. Immediately prior to infection, cells were washed twice with phosphate buffered saline (PBS) to remove debris. Cells were infected at an MOI of 100 in RPMI (3% FCS, 1% l-glutamine) and infection allowed to proceed for 1 h before three washes with RPMI (3% FCS, 1% l-glutamine, 50 μg/ml gentamycin). Cells were then incubated in the same media until harvest. For colony forming unit (CFU) counts, cells were washed three times with PBS before 200 μl of 1% Triton X-100 (Sigma) in PBS was added to each well to lyse cells. Bacteria were serially diluted in LB broth and spread onto LB agar plates containing the appropriate antibiotic. Total bacteria were enumerated by CFU counts after overnight incubation at 37 °C.

### Gram-staining and bacterial cell length measurement

Gram-staining was carried out using an Analytical Gram-Stain Kit (Fluka) and bacteria imaged on a Leica DM2000 microscope. Three biological replicates were imaged, with at least 10 images per slide being taken. Cell length calculation was performed using the Measurement PlugIn on ImageJ (National Institutes of Health).

### Fluorescent imaging of bacteria

Bacterial strains were grown overnight at 37 °C before back dilution the following morning and growth further to an OD_600_ of ~0.6. One millilitre of culture was then centrifuged at 8000 × *g* for 3 min, washed twice in PBS before being fixed in 4% (w/v) paraformaldehyde (PFA) at room temperature, for 15 min. Samples were washed twice more in PBS before being dried onto coverslips and mounted onto glass slides with 4′,6-diamidino-2-phenylindol (DAPI)-containing mounting media (VWR). Images were taken using a Leica DMi8 fluorescent microscope. At least three biological replicates were imaged for each strain, with at least ten images per coverslip. Images were analysed using the CellCounter PlugIn on ImageJ.

### Fluorescent imaging of bactofection

Following infection of coverslip-seeded MDA-MB-231 cells, or transfection with 1 mg of purified pEGFP DNA using Lipofectamine® 2000, cells were washed with PBS, fixed by adding 4% (w/v) PFA and incubated at room temperature for 15 min. Samples were washed with 1 ml PBS and stored in PBS until immunofluorescent staining. The cells were washed three times for 3 min with PBS and stained with phalloidin-rhodamine (1 U/sample, ThermoFisher) and 300 nM DAPI (diluted in PBS) for 20 min. Samples were washed three times with PBS. A drop of Vectashield Mounting Medium (Vector Laboratories) was placed on the surface of a microscope slide and the coverslip inverted and sealed on top of the slide using clear nail polish. Images were acquired using a Leica DMi8 for standard fluorescence microscopy. GFP expression cut-offs were set against uninfected cells. GFP positive cells were counted in at least ten images per coverslip in three independent biological replicates.

### Immunoblot analysis

For immunoblot assays bacterial strains were grown as previously but with mitomycin-C treatment (5 μg/ml, Sigma) for 4 h at 30 **°**C, 120 rpm. Cells were harvested in late log phase, washed twice in PBS, before being centrifuged and subjected to a freeze-thaw cycle. Cells were re-suspended in bacterial lysis buffer (50 mM Tris pH 8.0, 10% v/v glycerol, 0.1% Triton X-100, 100 μg/ml lysozyme, 3 U/ml DNAseI, 2 mM MgCl_2_, cOmplete^TM^ Mini (EDTA-free Protease Inhibitor Cocktail [Sigma]), before being sonicated three times at 10 mAmps for 30 s, on ice. Cells were lysed by five passages through a syringe using a 26-gauge needle before centrifugation at 16,000 × *g*. Protein concentrations were determined using a Pierce BCA Protein Assay Kit (ThermoFisher Scientific) before Western blotting was carried out using antibodies against either RecA (Abcam, ab63797) or GroEL (Abcam, ab90522).

### Data analysis

All values are expressed as the mean±standard error of the mean (SEM). Statistical analyses were performed using a Student’s *t*-test or one-way analysis of variance (ANOVA) with Tukey multiple comparisons test as detailed in each figure legend, to compare differences between groups. All statistical analyses were performed using GraphPrism 5 (GraphPad Software, Inc., CA, USA). Differences between groups were considered significant when *p* < 0.05.

## Results

### *S.* Typhimurium transformed with pEGFP displays a filamentous phenotype

In order to assess the bactofection capability of *S*. Typhimurium strain SL7207 it was transformed with the eukaryotic reporter plasmid, pEGFP (SL-pEGFP), which encodes *EGFP* under the control of the cytomegalovirus (CMV) enhancer/promoter region [43]. Transcription of *EGFP* via this plasmid is restricted to eukaryotic cells, so GFP signal is only evident following bactofection of pEGFP into recipient host cells.

**Fig. 1:**
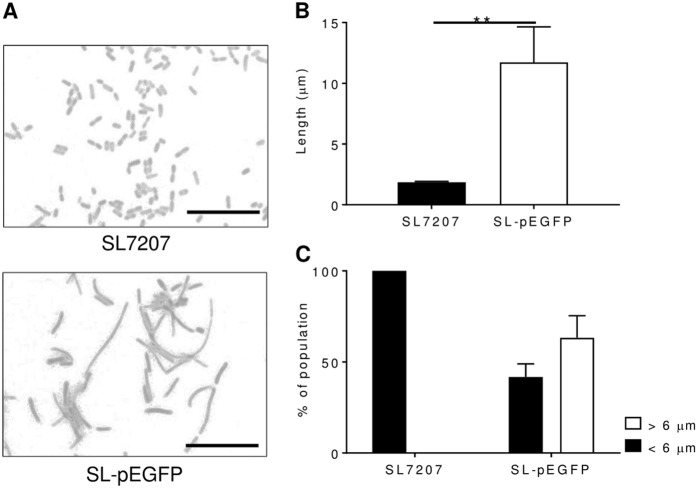
Morphology of SL7207-pEGFP. Wild type SL7207 and SL-pEGFP were grown in culture to mid-log phase, Gram-stained and examined by light microscopy. Representative light microscopy images of Gram-stained SL7207 cultures, both wild type and SL-pEGFP (**a**). Scale bars 10 µm. The mean length of individual bacteria (**b**). Quantification of the proportion of SL7207 in culture which were filamentous (>6 µm; **c**). Results displayed are the average of at least two independent biological replicates. Error bars SEM. Representative Gram-stains are shown for each culture. Statistical analyses performed using a Students *t* test where *p* < 0.01**

Bactofection in multiple tissues has been reported for *S*.Typhimurium and the SL7207 strain has been demonstrated to be highly capable of delivering DNA to mammalian cells [36, 23, 44]. Light microscopic analysis however highlighted an unusual morphological feature of transformed strains with SL-pEGFP cultures displaying a filamentous phenotype unlike wild type SL7207 cultures (Fig. 1a). The mean cell length of SL-pEGFP was significantly greater than SL7207 (Fig. 1b), but interestingly, not all bacteria within the culture displayed a filamentous phenotype, which has previously been defined as a cell length of more than 6 μm [45]. The proportion of SL-pEGFP which were filamentous was 39.58% (±10.82%), whereas there were <0.01% of SL7207 which were filamentous (Fig. 1c).

**Fig. 2:**
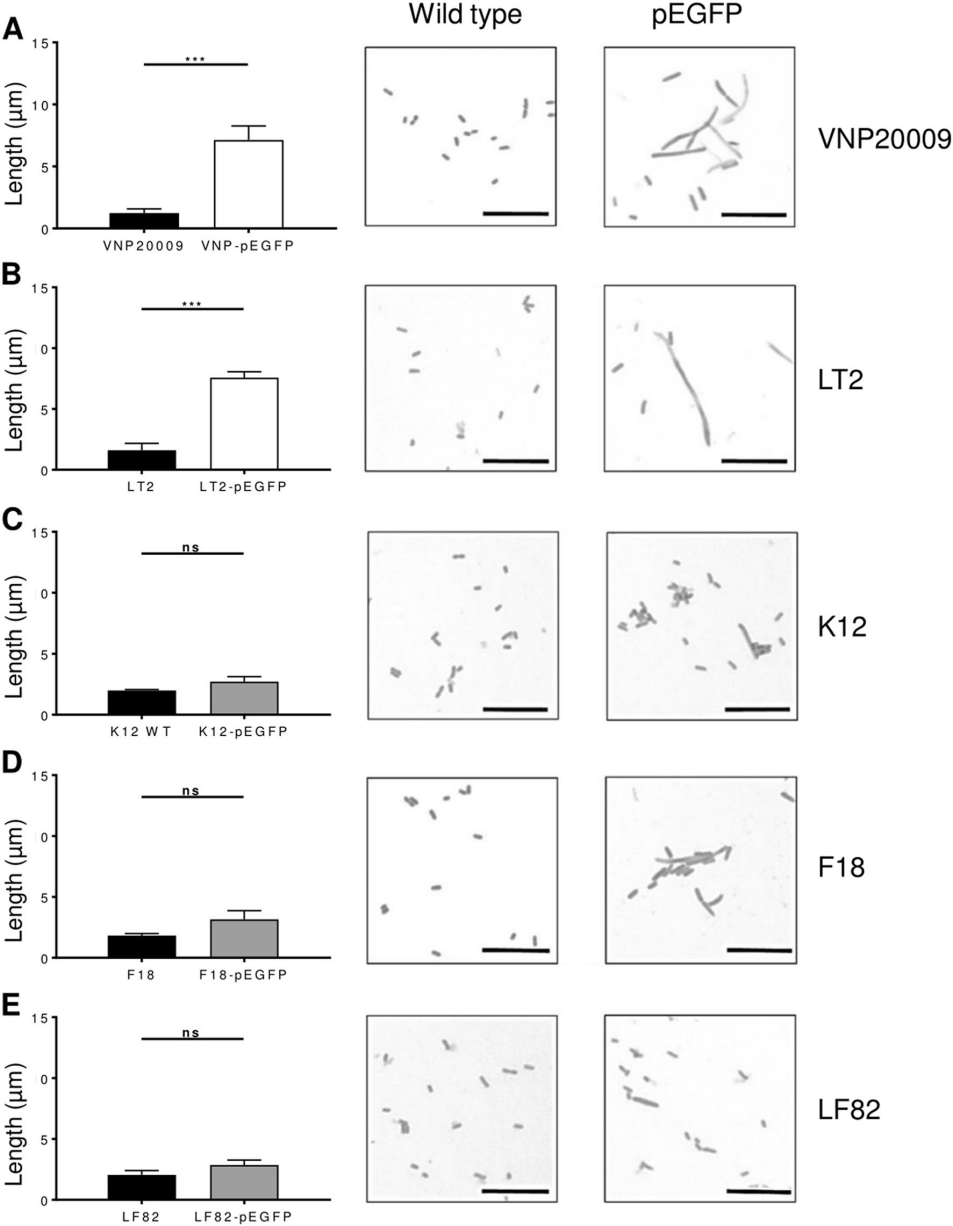
Morphology of *S*. Typhimurium and *E. coli* strains transformed with pEGFP. The *S*. Typhimurium (**a** VNP20009 and **b** LT2) and *E. coli* (**c** K12, **d** F18, and **e** LF82) strains were transformed with pEGFP. Quantification of the mean cell lengths of cultures along with representative light microscopy images of Gram-stained wild type and pEGFP transformed cultures are shown, as indicated. Scale bars 10 µm. Results displayed are the average of three independent biological replicates. Error bars SEM. Representative Gram-stains are shown for each culture. Statistical analyses performed using a Students *t* test where *p* < 0.001***; ns not significant

The filamentous phenotype induced by the transformation of pEGFP into *S*. Typhimurium was not restricted to SL7207 as multiple other *S*. Typhimurium strains tested also displayed filamentous phenotypes following transformation with pEGFP, including another cancer targeting strain VNP20009 and the common lab strain *S*. Typhimurium, LT2 (Fig. 2a, b). The observed differences between these pEGFP-transformed *S*. Typhimurium strains and their untransformed counterparts were comparable to those seen with SL7207.

### Effects of pEGFP transformation on *E. coli* strains

To investigate whether this was a *S*. Typhimurium-specific phenomenon, *E. coli* strains were transformed with pEGFP and the mean cell lengths were compared to untransformed, wild type cultures, as before. Although there was a slight increase in the average mean cell lengths of the transformed cultures, there were no statistically significant differences between pEGFP-transformed cultures and non-transformed wild-type cultures for the laboratory *E. coli* strain K12 (Fig. 2c; *p* = 0.2269), commensal *E. coli* F18 (Fig. 2d; *p* = 0.2070) or pathogenic *E. coli* LF82 (Fig. 2e; *p* = 0.2715). These data suggested that pEGFP-induced filamentation was *S*. Typhimurium-specific.

### Effects of filamentation on SL-pEGFP growth and invasion of MDA-MB-231 cells

**Fig. 3:**
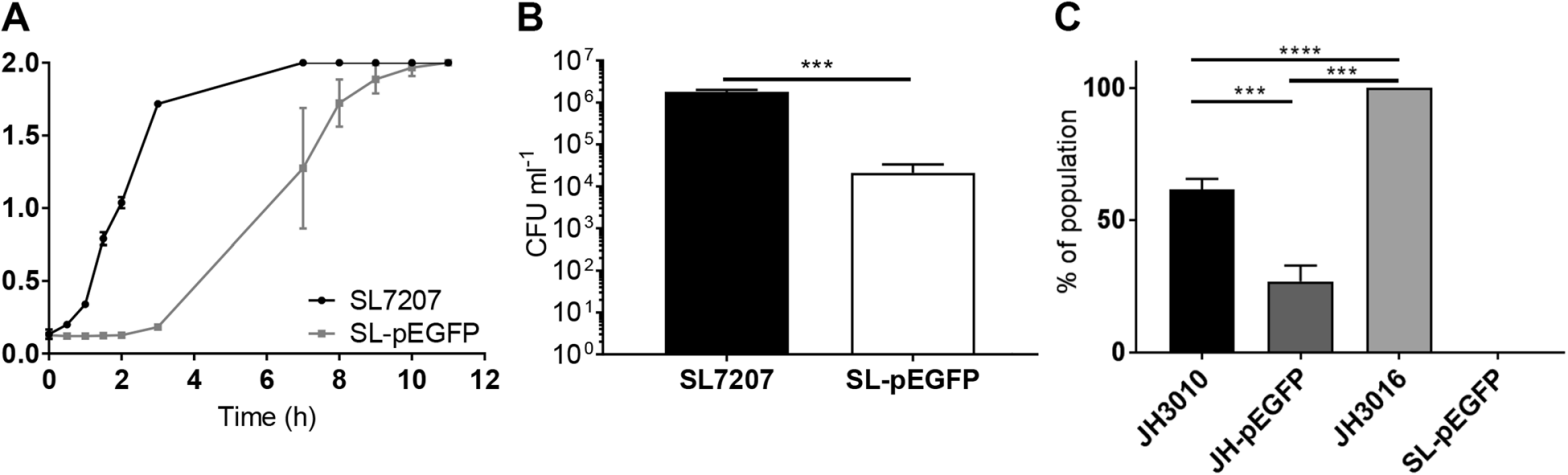
Growth and invasion characteristics of SL-pEGFP. Wild type SL7207 and SL-pEGFP were compared for growth rate (**a**) and capacity to invade MDA-MB-231 tumour cells (**b**). CFU counts of SL7207 and SL-pEGFP recovered from MDA-MB-231 cells at 2 h post infection. (**c)** Quantification of the proportion of bacteria in culture expressing SPI-1 at mid-log phase using the SPI-1 reporter strain, JH3010. JH3016 served as a positive control as it is a constitutively expressing GFP strain. SL-pEGFP served as a negative control for GFP expression as *EGFP* was under the control of the CMV promoter. Results displayed are the average of three independent biological replicates. Error bars SEM. These experiments were conducted in liquid culture. Statistical analyses performed using a Students *t* test (**b**) or One Way Anova (**c**) where *p* < 0.05*, *p* < 0.01**. *p* < 0.001***, *p* < 0.0001****

In order to assess the effects that pEGFP-induced filamentation had on the behaviour of *S*. Typhimurium, multiple phenotypic characteristics of SL-pEGFP were compared to SL7207. SL-pEGFP displayed slower cell growth in vitro compared to SL7207 (Fig. 3a), as well as decreased capacity to invade MDA-MB-231 cells (Fig. 3b).

The decreased virulence of SL-pEGFP was further investigated by examining induction of *Salmonella* Pathogenicity Island-1 (SPI-1), a key set of virulence determinants that mediate invasion of eukaryotic cells [2, 46, 47]. Using *S*. Typhimurium JH3010, a *prgH-GFP* reporter strain, we determined induction of *prgH* which encodes a type 3 secretion system (T3SS) needle apparatus protein essential for SPI-1-mediated invasion of intestinal epithelial cells [48]. JH3010 and SL7207 are both derived from wild type *S*. Typhimurium SL1344, so SL1344 transformed with pEGFP (SL-pEGFP) was used as a negative control to ensure any GFP signal was coming from expression of plasmid-borne *EGFP*. It was found that JH3010-pEGFP had decreased *prgH-GFP* expression compared to JH3010 (Fig. 3c), indicating a decrease in SPI-1 expression which was likely a contributory factor in the attenuated invasion capacity of *S*. Typhimurium-pEGFP.

### Induction of stress responses in pEGFP-transformed S.

**Fig. 4:**
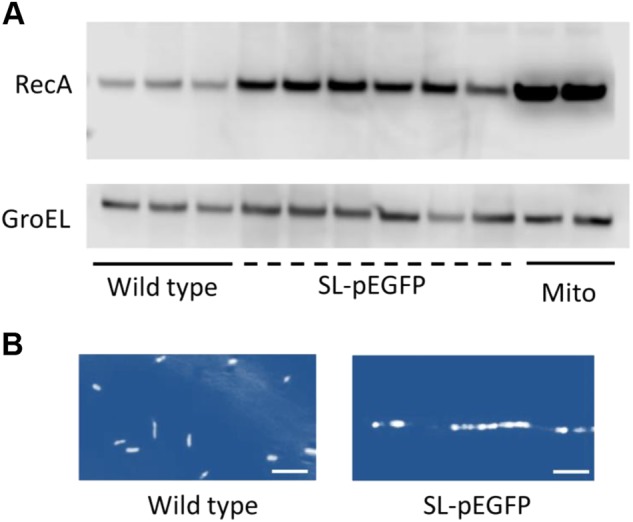
Stress response activation in *S*. Typhimurium carrying pEGFP. Cultures were grown to mid-log phase (OD_600_ of ~0.6) and harvested for western blot analysis for stress response proteins or immunofluorescence staining. **a** Immunoblot analysis for RecA across multiple wild type or pEGFP-transformed SL7207 colonies with mitomycin C-treated (Mito) wild type SL7207 cultures serving as a positive control for RecA activation. RecA and GroEL levels were determined in at least three independent replicates of SL7207 and SL-pEGFP. **b** Representative DAPI-stained wild type and SL-pEGFP cultures. Images were pseudo-coloured to aid visualisation of the segmented filamentous phenotype of SL-pEGFP. Scale bars 5 µm. Representative DAPI-stained images are shown for each culture

Filamentation is intimately linked with cell stress so the role of the stress response in pEGFP mediated filamentation was investigated [45, 49]. The SOS DNA damage stress response has been investigated in filamentation studies and so was further investigated here [50, 51]. The SOS stress response protein, RecA was upregulated in SL*-*pEGFP, suggesting that the SOS response is increased in SL-pEGFP cultures (Fig. 4a).

The induction of the SOS response inhibits septation of replicating bacteria [52, 49]. This phenotype was investigated in the filamentous cultures by staining fixed cultures with the nuclear stain DAPI. Fluorescence microscopy images of filamentous bacteria demonstrated multiple nuclei aligned along a filamentous bacterium, a phenotype not evident in the wild type cultures. These data further suggested a role for inhibited septation and the SOS response in the SL-pEGFP cultures (Fig. 4b).

### The contribution of the f1 *ori* in pEGFP to the filamentous phenotype of SL-pEGFP

**Fig. 5:**
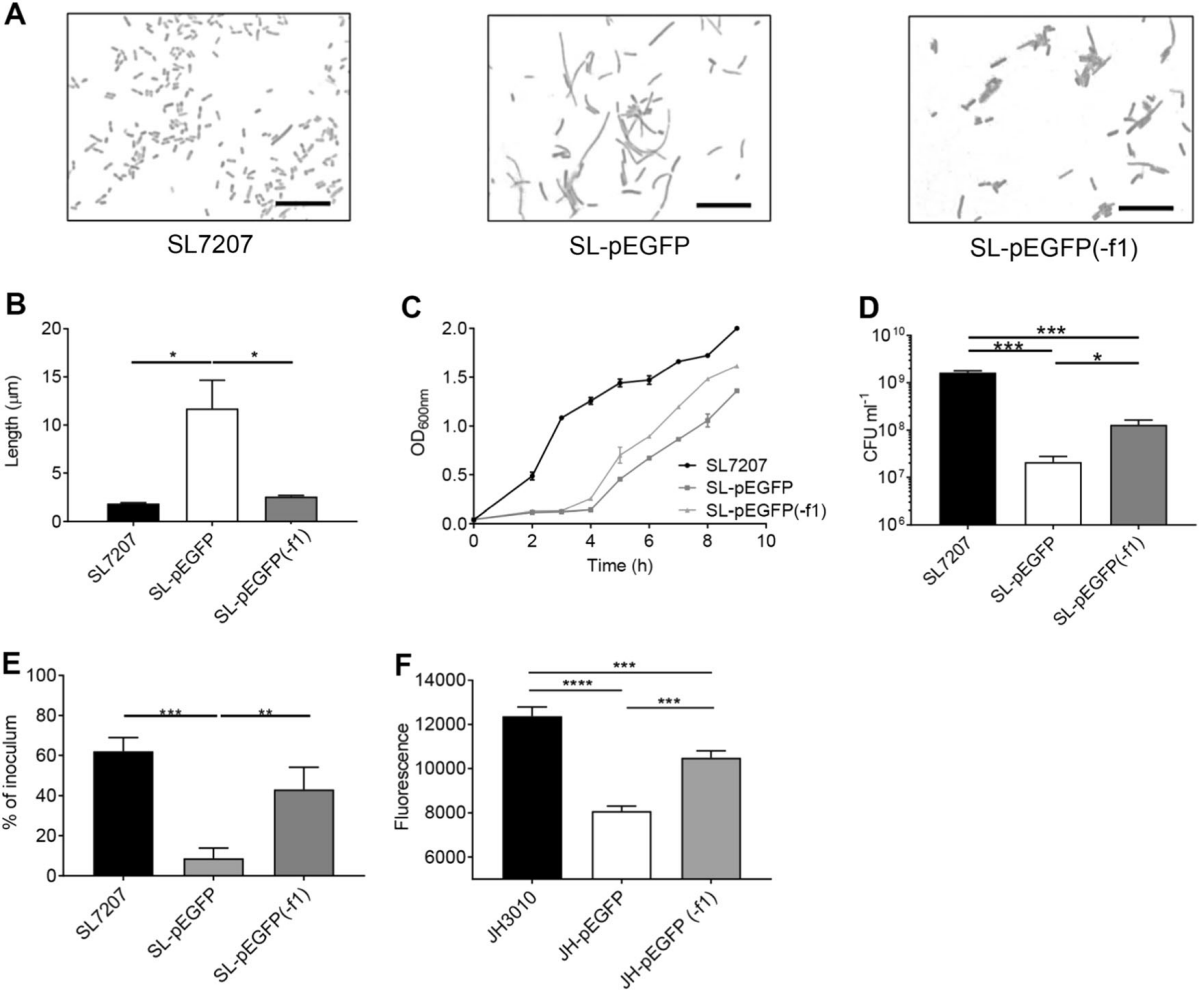
Removal of the f1 *ori* removes the filamentous phenotype and improves invasion. Wild type SL7207, SL-pEGFP and SL-pEGFP(-f1) were compared for morphology (**a**); average length (**b**); growth rate (**c**); biomass at OD_600nm_ 0.6 (**d**); and capacity to invade MDA-MB-231 tumour cells (**e**). CFU counts of SL7207 and SL-pEGFP recovered from MDA-MB-231 cells at 2 h post infection. **f** Quantification of bacterial invasion through SPI-1 expression using the SPI-1 reporter strain, JH3010. JH3010 was transformed with pEGFP and pEGFP(-f1), respectively. Error bars SEM. Statistical analyses performed using One Way Anova where *p* < 0.05*; *p* < 0.01**; *p* < 0.001***. Results displayed are the average of three independent biological replicates

It was hypothesised that there was a feature of pEGFP which was responsible for inducing this phenotype. The SOS response is triggered in *S*. Typhimurium in response to the presence of cytoplasmic single stranded DNA (ssDNA). Investigation of the pEGFP plasmid revealed the presence of filamentous phage 1 origin of replication (f1 *ori*), a phagemid capable of phage-directed ssDNA production [53]. Phages have been reported to induce the SOS response in *S. enterica* [54]. Therefore, it was hypothesised that the f1 *ori* may be responsible for inducing the filamentous phenotype in *S*. Typhimurium-pEGFP. To test this hypothesis, the f1 *ori* in pEGFP was replaced with *lacZ* from pUC19 to give rise to pEGFP(-f1), which no longer contained the f1 *ori*, but maintained the functional elements to enable bactofection (Supplementary Fig. S1). This plasmid was then transformed into SL7207 (SL-pEGFP(-f1)) and the morphology of the bacteria was assessed. Light microscopy imaging of Gram-stained SL-pEGFP(-f1) demonstrated that this plasmid did not induce a filamentous phenotype (Fig. 5a). The mean cell lengths of the wild type and SL-pEGFP(-f1) were significantly different from those of SL-pEGFP cultures (Fig. 5b). Growth rate was also increased in SL-pEGFP(-f1) compared to SL-pEGFP, while at OD_600_ of 0.6 there was a significant increase in recovery of CFUs from SL-pEGFP(-f1) cultures indicating that filamentation had decreased compared to SL-pEGFP (Fig. 5c, d). Finally, there was a significant increase in invasion of MDA-MB-231 cells by the SL-pEGFP(-f1) strain indicating that virulence of this strain had been restored by replacement of the f1 *ori* (Fig. 5e). As shown previously (Fig. 3c), transformation of pEGFP into JH3010, a SPI-1 reporter strain resulted in decreased SPI-1 expression. Subsequently, removal of the f1 *ori* restored SPI-1 expression to near wild type JH3010 levels (Fig. 5f). Taken together this data suggested that the f1 *ori* was responsible for inducing the filamentous phenotype in the *S*. Typhimurium cultures and that its replacement could abrogate this effect.

To further demonstrate the role of the f1 *ori*, an alternative f1 *ori* containing plasmid, pLuc, was investigated alongside plasmids which determined any potential roles for other features of pEGFP including; the *EGFP* transgene, the plasmid *ori* and the antibiotic resistance cassette. These features had been suggested to induce bacterial stress which may lead to filamentation [55–57]. However, only bacteria carrying an f1 *ori* containing plasmid were filamentous with pLuc containing bacteria displaying both a similar phenotype and cell length to SL-pEGFP (Supplementary Fig. S2).

**Fig. 6:**
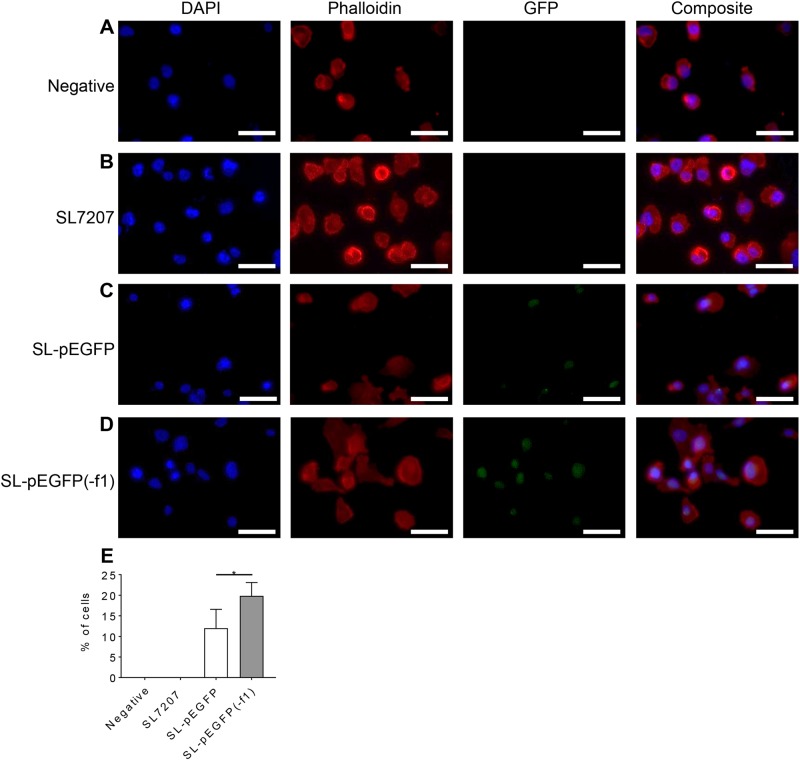
Removal of the f1 *ori* increases the bactofection capacity of *S*. Typhimurium-pEGFP. MDA-MB-231 cells were treated with PBS (**a**); or infected with SL7207 (**b**); SL-EGFP (**c**); or SL-pEGFP(-f1) (**d**) at an MOI of 100:1, for 2 h before fixation with 4% PFA. Subsequently, cells were immunostained using DAPI to stain nuclear material and rhodamine-phalloidin to stain the actin cytoskeleton. Percentage of GFP positive cells were quantified in ten individual images across three biological replicates (**e**). Scale bars 50 µm. Statistical analyses performed using One Way Anova where *p* < 0.05*

Lastly, to ensure that removal of the f1 *ori* improved the bactofection potential of SL-pEGFP(-f1), MDA-MB-231 cells were again infected with SL72027, SL-pEGFP and SL-pEGFP(-f1). Increased bactofection was noted in cells infected with SL-pEGFP(-f1) (19.8 ± 3.25% Standard deviation) compared to SL-pEGFP (12 ± 4.58% Standard deviation) (Fig. 6). This increase indicated that the f1 *ori* was responsible for the reduction in SL-pEGFP bactofection.

## Discussion

The use of bacteria as gene delivery vehicles is an area of growing research interest with bacterial genera such as *Escherichia, Listeria*, and *Salmonella* all capable of carrying and delivering genetic material to target cells [33–36]. The ability of bacteria to deliver genetic material to eukaryotic systems has been demonstrated in vivo in multiple pathologies including cystic fibrosis and cancer [11, 19, 20, 22, 24, 43]. While bactofection as a potential therapeutic strategy increases in popularity, the exact mechanisms underlying its success in delivering genetic material to host cells are still unclear [30, 31]. Of all of the bacterial species used for bactofection that have been reported in the literature, *Salmonella* spp. are arguably the best characterised. *Salmonella* has been employed to deliver a variety of eukaryotic genes to tumours in vivo including apoptosis-associated genes Second mitochondrial derived activator of caspases *(SMAC)* and TNF-related apoptosis inducing ligand (*TRAIL*), as well as for cytokine gene therapy in subcutaneous tumour mouse models [11, 18, 37, 38]. These transgenes are borne on plasmids that can be released for uptake upon entry into the target mammalian cell. While these plasmids have been investigated in an attempt to understand the drivers behind mammalian cell uptake of the bacterial carried DNA, as we demonstrate here the makeup of these plasmids can also seriously impact the bactofection capability of *S*. Typhimurium.

For this study, *S*. Typhimurium strains were transformed with the eukaryotic expression vector, pEGFP, a plasmid previously reported as being capable of being transferred into eukaryotic cells, resulting in subsequent *EGFP* expression [43, 58, 59]. However, our initial experiments found that SL-pEGFP induced a filamentous phenotype that was apparent at high magnification (Fig. 1a). This phenotype was apparent in both cancer targeting bactofection strains (SL7207 and VNP20009) and wild type *S*. Typhimurium (LT2) and did not appear to be strain specific (Fig. 2a, b). We could find no prior reports of a eukaryotic expression vector inducing filamentation in bacteria. It has been reported that certain mutations in a pBR332 plasmid can induce invasion defects in *S*. Typhimurium, but this was attributed to plasmid architecture [57]. The degree of filamentation was quite striking (Fig. 1c) with the mean length of the transformed SL7207 cultures more than three times that of the wild type. Additionally 39.58% (±10.82% standard deviation) of each culture could be classed as filamentous based on a previous classification system whereby bacteria greater than three cell lengths (6 µm) were determined to be filamentous [45]. A factor likely to be critical to the lack of prior reporting of this phenotype is that *E. coli* strains transformed with pEGFP did not exhibit filamentation (Fig. 2c–e). Given the majority of cloning work with mammalian vectors such as pEGFP occurs in *E. coli* laboratory strains, such as the *E. coli* K12 strain which did not display a filamentous phenotype here, this may explain why this phenomenon went unnoticed. Filamentation was also absent in commensal and pathogenic *E. coli* strains indicating that immunity to filamentation again appears to occur across a wide range of *E. coli*.

Given the widespread use of pEGFP, the potential for this phenotype to be linked to other plasmids was investigated further. Our analysis led us to focus on the f1 *ori* a feature of pEGFP and common to plasmids such as pBluescript, pGEM and pcDNA3.1. Plasmids containing the f1 *ori* induced filamentation in *S*. Typhimurium while those without it exhibited no filamentous phenotype (Supplementary Fig. S2). To our knowledge this phenotype has not previously been reported with f1 *ori*-containing plasmids. The purpose of the f1 *ori* in the pEGFP plasmid is to facilitate ssDNA replication and phage packaging [53, 60]. The f1 *ori* is therefore likely an artefact due to the prior use of eukaryotic expression vectors as means to introduce mutations into genes on the vector upon induction with the appropriate phage. To activate the f1 *ori*, it must first be nicked by an endonuclease, Gp2 protein of the filamentous phage which recognises a consensus sequence in the origin sequence and cleaves a single strand to allow the initiation of ssDNA packaging by the phage [61]. Therefore, the presence of the f1 *ori* may lead to the generation of ssDNA by the Gp2 protein associated with the filamentous phage elements in *S*. *typhimurium*. Crucially the production of ssDNA is sufficient to activate the SOS stress response as seen here in SL-pEGFP. This stress response, induced by the cleavage of the LexA repressor, then allows for the SOS transcriptional programme to be activated [62]. The culmination of this transcriptional programme is the arrest of FtsZ oligomerisation and septation during cell division [63]. Septational arrest subsequently results in filamentation, with nuclear staining clearly depicting multiple nuclei along a single filamentous bacterium, as seen in SL-pEGFP (Fig. 4).

To overcome filamentation in SL-pEGFP we substituted the f1 *ori* with the lacZ gene (Supplementary Fig. S2), significantly reducing the filamentous phenotype. After f1 *ori* removal bacteria carrying the new plasmid became increasingly invasive and a significant increase in bactofection of MDA-MB-231 cells was observed (Fig. 6). While other constituents of the plasmid, such as the transgene, other *ori*s and resistance cassettes, were also examined none were seen to induce the filamentous phenotype. These have previously been implicated in stress induction in bacteria but none were seen to be involved in the phenotype described here [55–57].

Given the f1 *ori* is common to many plasmids, the results described here have important implications for future studies where bacterial carriage of plasmids is required, and for past studies where such plasmids have been used for bactofection. Overall, this study indicates that while plasmids are a crucial tool in manipulating and equipping bacteria for various purposes, the burden they can often place on bacterial fitness, and the effects this may have on resulting data and its interpretation, are still incompletely understood.

## Electronic supplementary material

Fig. S1

Fig. S2

Supplemental legends
