# Author Correction: Increasing the bactofection capacity of a mammalian expression vector by removal of the f1 *ori*

**DOI:** 10.1038/s41417-019-0086-x

**Published:** 2019-02-20

**Authors:** Síle A. Johnson, Michael J. Ormsby, Anne McIntosh, Stephen W. G. Tait, Karen Blyth, Daniel M. Wall

**Affiliations:** 1 Institute of Infection, Immunity and Inflammation, College of Medical, Veterinary and Life Sciences, Sir Graeme Davies Building, University of Glasgow, Glasgow, G12 8TA UK; 2 Cancer Research UK Beatson Institute, University of Glasgow, Garscube Estate, Switchback Road, Glasgow, G61 1BD UK

**Correction to:** **Cancer Gene Therapy** (2018);

10.1038/s41417-018-0039-9;

published on 13 August 2018.

This article was originally published under Nature Research’s License to Publish, but has now been made available under a [CC BY 4.0] license. The PDF and HTML versions of the article have been modified accordingly.

