# Supplementary figures and images for: Increasing the bactofection capacity of a mammalian expression vector by removal of the f1 ori
Source: Cancer Gene Ther. 2018 Aug 13;26(7):183–94. doi: 10.1038/s41417-018-0039-9 (PMC6760541; doi:10.1038/s41417-018-0039-9)

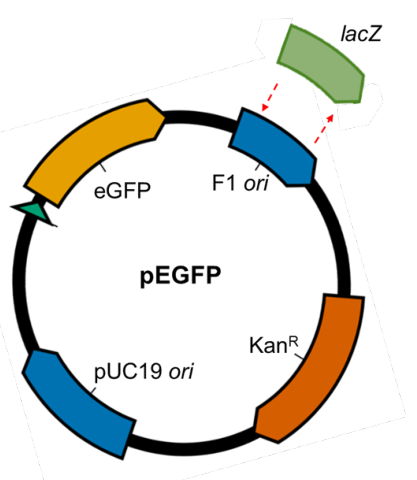

Supplement: Supplementary file 1 — Fig. S1 [file 41417_2018_39_MOESM1_ESM.tif]

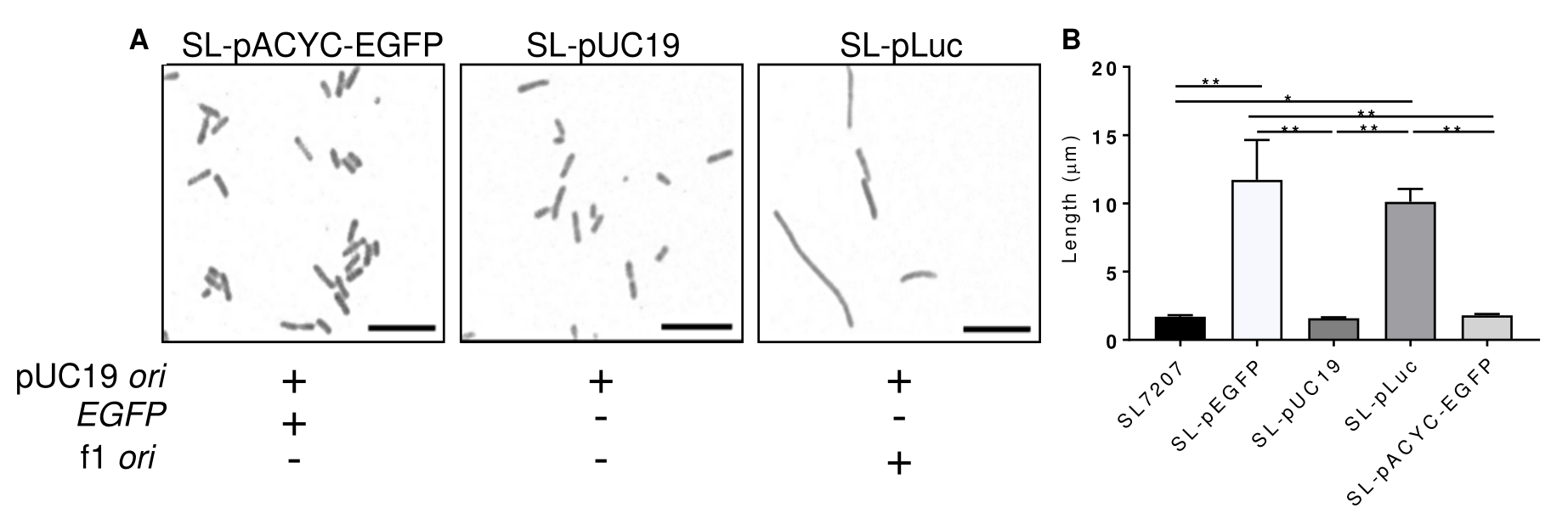

Supplement: Supplementary file 2 — Fig. S2 [file 41417_2018_39_MOESM2_ESM.tif]
