# Supplementary material for: Increasing the bactofection capacity of a mammalian expression vector by removal of the f1 ori
Source: Cancer Gene Ther. 2018 Aug 13;26(7):183–94. doi: 10.1038/s41417-018-0039-9 (PMC6760541; doi:10.1038/s41417-018-0039-9)
Supplement: Supplementary file 3 — Supplemental legends [file 41417_2018_39_MOESM3_ESM.docx]

**Supplementary Figure Legends**

**Fig. S1 f1 *ori* replacement in pEGFP.**

The f1 ori was replaced in pEGFP with lacZ.

**Fig. S2 Effect of specific plasmid features on the pEGFP-induced filamentous phenotype.** The f1 *ori* was investigated for its ability to induce filamentation in pEGFP-transformed *S*. Typhimurium. Representative light microscopy images of Gram-stained SL7207, transformed with pACYC-EGFP, pUC19 or pLuc are shown (A). The mean cell lengths of the cultures were quantified and compared with SL7207 and SL-pEGFP (B). Presence or absence of the plasmid features pUC19 *ori*, eGFP and f1 *ori* are indicated by ‘+’ and ‘-.’ Scale bars 10 µm. Statistical analysis was performed using a One Way ANOVA with p < 0.05*; p < 0.01**.
